# Shape and Enhancement Analysis as a Useful Tool for the Presentation of Blood Hemodynamic Properties in the Area of Aortic Dissection

**DOI:** 10.3390/jcm9051330

**Published:** 2020-05-02

**Authors:** Andrzej Polanczyk, Aleksandra Piechota-Polańczyk, Ludomir Stefanczyk, Michal Strzelecki

**Affiliations:** 1 The Main School of Fire Service, 01-629 Warsaw, Poland; 2 Department of Medical Biotechnology, Jagiellonian University, 30-387 Krakow, Poland; aleksandra.piechota-polanczyk@uj.edu.pl; 3 Department of Radiology and Diagnostic Imaging, Medical University of Lodz, 90-153 Lodz, Poland; ludomir.stefanczyk@umed.lodz.pl; 4 Institute of Electronics, Lodz University of Technology, 93-005 Lodz, Poland; michal.strzelecki@p.lodz.pl

**Keywords:** aortic dissection, brightness analysis, enhancement analysis, image processing

## Abstract

The aim of this study was to create a mathematical approach for blood hemodynamic description with the use of brightness analysis. Medical data was collected from three male patients aged from 45 to 65 years with acute type IIIb aortic dissection that started proximal to the left subclavian artery and involved the renal arteries. For the recognition of wall dissection areas Digital Imaging and Communications in Medicine (DICOM) data were applied. The distance from descending aorta to the diaphragm was analyzed. Each time Feret (D_F_) and Hydraulic (D_Hy_) diameter were calculated. Moreover, an average brightness (B_AV_) was analyzed. Finally, to describe blood hemodynamic in the area of aortic wall dissection, mathematical function combining difference in brightness value and diameter for each computed tomography (CT) scan was calculated. The results indicated that D_F_ described common duct more accurately compare to D_Hy_. While, D_Hy_ described more accurately true and false ducts. Each time when connection of true and false duct appeared, true duct had lower brightness compare to common duct and false duct. Moreover, false duct characterized with higher brightness compare to common duct. In summary, the proposed algorithm mimics changes in brightness value for patients with acute type IIIb aortic dissection.

## 1. Introduction

Aortic dissection with tear formation in the inner lining of the aorta, is one of the worst cardiovascular emergencies, associated with considerable morbidity and mortality [1,2,3]. The type of aortic dissection is characterized with the use of Stanford classification [4]. Depending on the location of the aortic dissection, one distinguishes type A at ascending aorta which typically requires surgical interventions, and type B at descending aorta due to often recurrence, progressive dilatation of lumen and aortic rupture [5,6]. Hemodynamic parameters are believed to play a crucial role in the formation and the progression of the aortic dissection [7]. The blood flow pattern within the dissected aorta is dominated by locally highly disturbed and possibly turbulent flow with strong recirculation [8].

Various mathematical models have been recently developed to understand cardiovascular system [6,9,10]. Among those are bioreactors describing mechanical properties of veins and artificial vessels [7,11,12]. Moreover, application of computational methods, including the computational fluid dynamic (CFD) technique in the topic of blood flow in vessels is widely described in the literature [13,14]. Navier–Stokes equations are applied for the description of three-dimensional blood hemodynamic [15]. Depending on the analyzed case, blood is considered as a Newtonian [16] or non-Newtonian fluid [17]. Moreover, the influence of blood hemodynamic on a vessel’s wall with the use of wall shear stress may be investigated with CFD technique [18]. Blood flow simulation provides important information, crucial for assessment of blood distribution for patients affected by vascular diseases, e.g., stenosis or aortic dissection [19,20]. Numerical methods as well as experimental devices require importing imaging [21,22]. Medical imaging techniques, e.g., computational tomography (CT) or magnetic resonance imaging (MRI), enable detail visualization of morphology of cardiovascular system dysfunction, e.g., aortic dissection [23]. Region growing or level set algorithms are applied for the vascular segmentation [24]. Furthermore, extraction of centerline for the vessel reconstruction is used [15]. However, none of the imaging techniques allow the prediction blood hemodynamic after surgical intervention [25,26,27]. The image processing of medical data allows the provision of realistic in vivo conditions for patient specific analysis, e.g., reliable anatomical 3D geometries of human cardiovascular system [28]. Thus, aortic dissection may be improved by a specific therapy and/or application of advanced prognostic tools, such as computer simulations [29]. Using this approach, it is possible to measure hemodynamic parameters within three-dimensional (3D) models that provide important information on hemodynamic changes within the true and false lumen. Moreover, changes in contrast enhancement with the use of contemporary computer tomography allow estimation of vessels’ narrowness [30,31]. In our study we have a reliable enhanced reference point, i.e., both channels filled after heart systole. Therefore, the differences occurring in them result only from different outflow conditions from the canals and not from conditions related to heart efficiency. The assessment of the width of the channels currently adopted is not perfect and there is a margin of patients in whom, despite good communication of the channels, dissection progresses and there are symptoms of organ ischemia. Therefore, the aim of this study was to create a tool for visualization of blood hemodynamic properties in the area of aortic dissection with the use of quantitative analysis of MRI data.

The paper is organized as follows: In Section II medical data, mathematical model, and its verification was described. Section III presents the results directed in the mathematical description of brightness value, aortic diameter and relation of both parameters. In Section IV a discussion was proposed while, Section V concludes the paper.

## 2. Experimental Section

Medical data was collected from three male patients aged from 45 to 65 years after CT angiography (CTA) (GE Light-Speed 64 VCT; GE Healthcare, Fairfield, CT, USA) who underwent treatment in the Barlicki Hospital No. 2 in Lodz (Poland) in 2016. During CTA patients obtained a contrast (Visipaque) at a constant value of 1.5 mL per 1 kg of body weight. All participants gave written informed consent to the study. The study protocol was approved by the local ethics committee at the Medical University of Lodz (approval no.: RNN/126/07/KE). Inclusion criteria comprise acute type IIIb aortic dissection that started proximal to the left subclavian artery and involved the renal arteries in each of the analyzed cases [32,33] (Table 1).

For the recognition of wall dissection areas Digital Imaging and Communications in Medicine (DICOM) data (512 × 512 × 270 voxels, in-plane resolution of 0.78 × 0.78 mm, slice thickness 0.8 mm) from the aforementioned patients with acute complicated type B dissection was applied as previously described [7,35]. Segmentation process included the following steps: (1) CT angiography data was manually adjusted for brightness to achieve the highest contrast between analyzed aorta and surrounding tissue; (2) the region growing technique to extract aorta from the background was applied; and (3) gaps were eliminated manually using the ImageJ software and its tool for morphological holes filling. The implemented segmentation region-growing technique provided quite accurate results, since the aorta gray levels differed significantly from the image background. When compared to manual segmentation performed by the radiologist (the reference method), the estimated aorta parameter values (area, diameter) did not differ more than 5%. To reconstruct 3D model of aorta after segmentation process, a rendering process was performed. Moreover, a quantitative analysis of CT angiography data was performed [36]. Therefore, two parameters were calculated: (1) Brightness intensity to noise (BI) as a quotient of aorta brightness intensity and noise value, and (2) contrast to noise ratio (CNR) as a quotient of subtraction of aorta brightness intensity and background brightness to noise value (Table 2).

The highest brightness intensity was calculated in Pixels by placing Region of Interest in the center of the area represented by analyzed aorta (reaching 80 mm^2^). This operation was performed for all slices for particular patient for common duct, false duct and true duct separately. The mean of these values was used for further calculations. While, image noise was calculated as standard deviation measured as well in pixels and calculated for 100 mm^2^ drawn in two different regions outside the patient body (left, and right sides).

Furthermore, to describe spatial configuration of analyzed aortas geometric parameters were used [6,37]. On each CT scan Feret (D_F_) [38] (Equation (1)) and Hydraulic (D_Hy_) [39] (Equation (2)) diameters were calculated. The D_F_ was calculated as an average value of horizontal and vertical diameter of analyzed object, while the D_Hy_ was calculated as a division of cross-section area of the flow in channel to the wetted perimeter of the aortic cross-section.

D_F_ = (D_v_ + D_h_)/2, (1)

where: D_F_—Feret diameter, (mm), D_v_—the highest distance between two points on the perimeter of analyzed aorta, calculated in vertical direction, (mm), D_h_—the highest distance between two points on the perimeter of analyzed aorta, calculated in horizontal direction, (mm).

D_Hy_ = 4P/A, (2)

where: D_Hy_—Feret diameter, (mm), P—cross-section area of blood flow, [mm^2^], A—wetted perimeter of blood flow cross-section, (mm).

With the use of Osirix software (Pixmeo SARL, Bernex, Switzerland) an average brightness value (B_AV_) (Equation (3)) of the area representing blood inside aorta was calculated. The distance from descending aorta to the diaphragm was analyzed.

B_AV_ = ∑ B_i_/∑ APixel, (3)

where: B_AV_—average brightness value, [Pixel], B_i_—brightness value for i Pixel, [pixel], ∑ APixeli—number of Pixels in the analyzed area, [–].

Finally, to describe blood hemodynamic in the area of aortic wall dissection, mathematical function combining difference in brightness values (Equation (4)) and diameter (Equation (5)) for each CT scan was calculated.

Dif B_AV_ = B_AV Duct_i_ − B_AV Duct_i_, (4)

where: Dif B_AV_—difference in brightness value, [–], B_AV Duct_i_—average brightness value for analyzed duct, [Pixel].

Dif D_HY/F_ = D_HY/F Duct_i_ − B_HY/F Duct_i_, (5)

where: Dif D_HY/F_—difference in diameter, [–], D_HY/F Duct_i_—average diameter for analyzed duct, (mm).

Statistical analysis was performed using Statistica 12.0 software (StatSoft, Tulsa, OK, USA). Data were presented as mean ± standard deviation (SD). Moreover, the Bland–Altman method was utilized to analyze the agreement between Feret diameter and Hydraulic diameter. Spearman’s correlation rho analysis was used in addition. Comparisons between analyzed groups were made using the U Mann–Whitney test after verifying normality and variance. Data were considered as significantly different when *p* < 0.05, unless otherwise noted.

## 3. Results

### 3.1. Diameter Analysis

**Figure 1:**
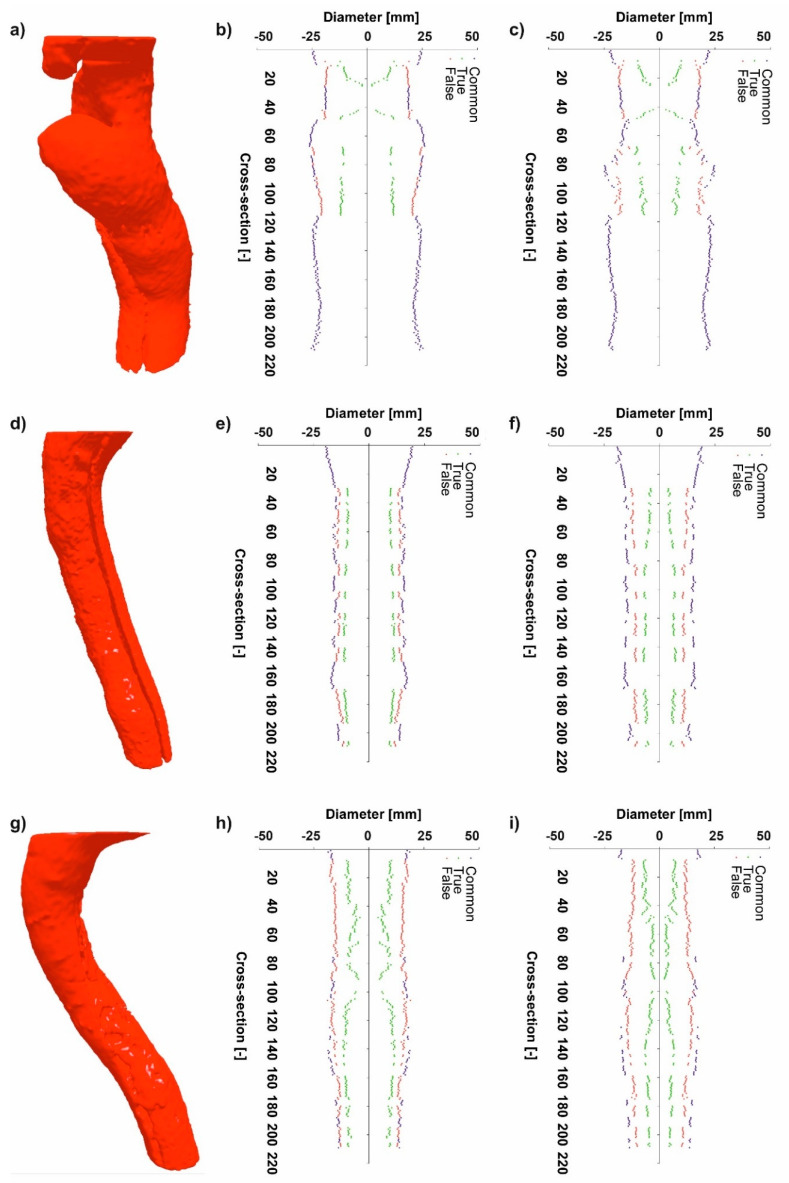
Spatial configuration of: (**a**) Patient 1 (P1), (**b**) D_F_ calculated for common, false and true duct for P1, (**c**) D_Hy_ calculated for common, false and true duct for P1, (**d**) patient 2 (P2), (**e**) D_F_ calculated for common, false and true duct for P2, (**f**) D_Hy_ calculated for common, false and true duct for P2, (**g**) patient 1 (P3), (**h**) D_F_ calculated for common, false and true duct for P3, and (**i**) D_Hy_ calculated for common, false and true duct for P3.

In the first step, to indicate the proper parameter to describe spatial configuration of analyzed domains, D_F_ and D_Hy_ for all three patients were analyzed. For patient number 1 (P1) (Figure 1a) average value of diameter for common duct was equal to 45.76 ± 3.70 mm and 41.21 ± 4.92 mm for D_F_ (Figure 1b) and D_Hy_ (Figure 1c), respectively. Moreover, average value of diameter for true duct was equal to 20.476 ± 5.18 mm and 15.71 ± 3.29 mm for D_F_ and D_Hy_, respectively. Furthermore, average value of diameter for the false duct was equal to 41.57 ± 4.23 mm and 35.47 ± 2.46 mm for D_F_ and D_Hy_, respectively.

**Figure 2:**
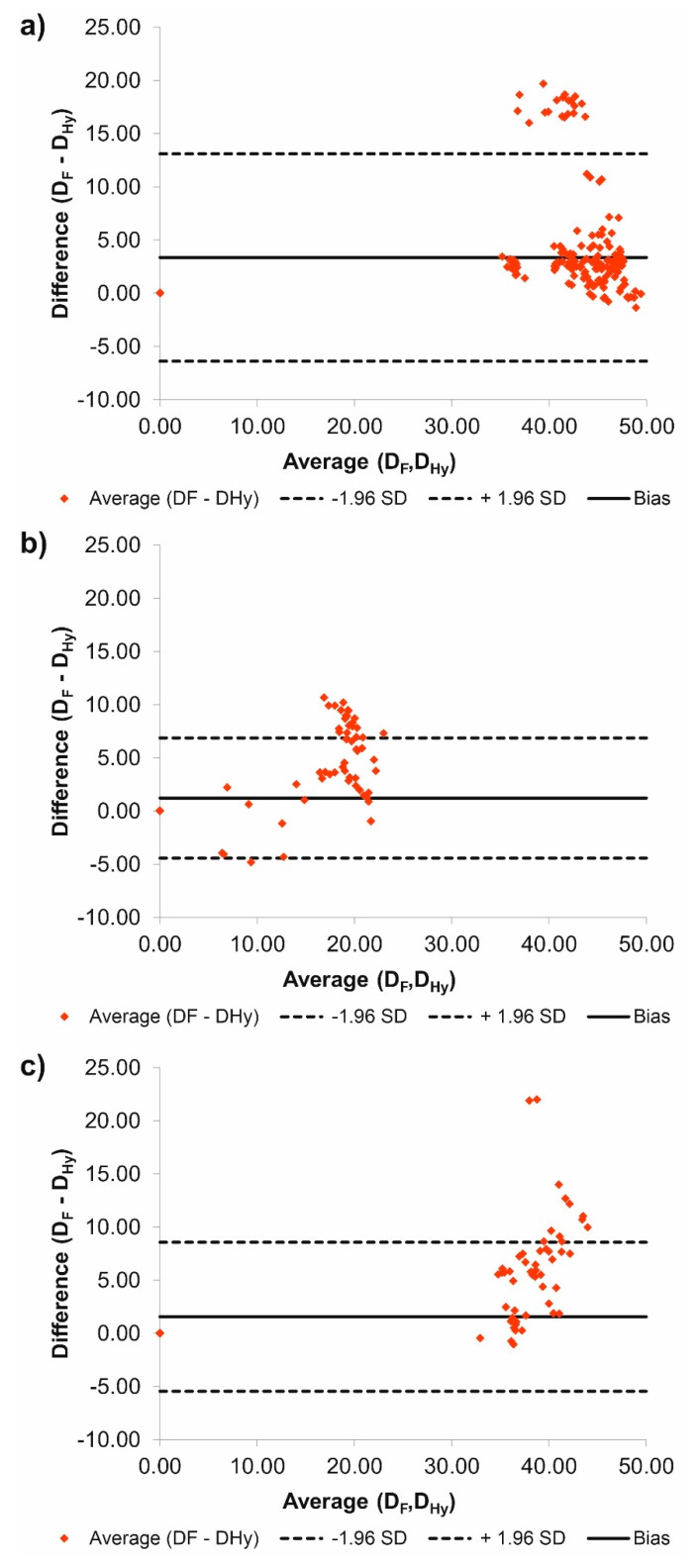
Comparison of D_F_ and D_Hy_ with the use of Bland-Altman analysis for: (**a**) Common duct, (**b**) true duct, and (**c**) false duct. For all analyzes *p* > 0.05.

Additionally, according to Bland–Altman analysis for the common duct for P1 the difference between Feret and Hydraulic diameter was equal to 3.35 mm for the range equal to 13.49 mm (Figure 2a), for the true duct it was 1.22 mm for the range 11.30 mm (Figure 2b), while for the false duct it was 1.55 mm for the range 14.02 mm (Figure 2c). In addition, for the common duct 19 points stand out the optimal range, for the true duct 20 points stand out the optimal range, while for the false duct 11 points stand out the optimal range.

The average cross-section for the common duct was equal to 1655.32 ± 258.03 mm and 1509.84 ± 244.77 mm for D_F_ and D_Hy_, respectively. The average cross-section for the true duct was equal to 346.50 ± 131.90 mm and 268.46 ± 83.92 mm for D_F_ and D_Hy_, respectively. While, average cross-section for the false duct was equal to 1358.24 ± 281.79 mm and 1139.17 ± 168.42 mm for D_F_ and D_Hy_, respectively.

For P2 (Figure 1d) average value of diameter for the common duct was equal to 31.91 ± 2.64 mm and 30.90 ± 2.78 mm for D_F_ (Figure 1e) and D_Hy_ (Figure 1f), respectively. The average value of diameter for the true duct was equal to 20.74 ± 1.31 mm and 11.85 ± 1.90 mm for D_F_ and D_Hy_, respectively. While average value of diameter for the false duct was equal to 27.14 ± 1.50 mm and 22.43 ± 1.44 mm for D_F_ and D_Hy_, respectively.

**Figure 3:**
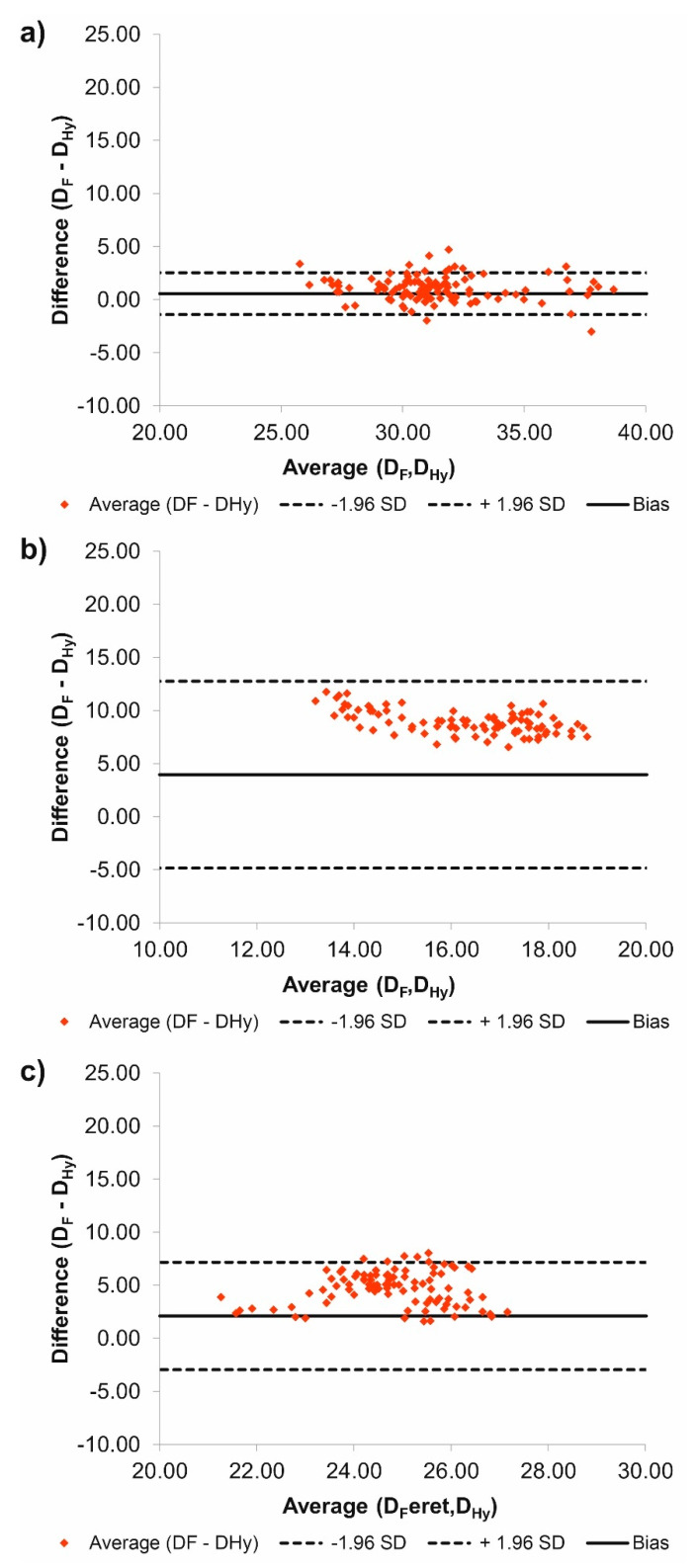
Comparison of D_F_ and D_Hy_ with the use of Bland–Altman analysis for: (**a**) Common duct, (**b**) true duct, and (**c**) false duct. For all analyzes *p* > 0.05.

Additionally, according to Bland–Altman analysis for the common duct for patient number 2 (P2) the difference between D_F_ and D_Hy_ was equal to 0.56 mm for the range equal to 3.94 mm (Figure 3a), for the true duct it was 3.95 mm for the range 17.60 mm (Figure 3b), while for the false duct it was 2.10 mm for the range 10.13 mm (Figure 3c). Moreover, for the common duct 11 points stand out the optimal range, for the true duct 0 points stand out the optimal range, while for the false duct 6 points stand out the optimal range.

The average cross-section for the common duct was equal to 805.31 ± 136.32 mm and 770.83 ± 135.93 mm for D_F_eret and D_Hy_draulic diameter, respectively. Moreover, average cross-section for the true duct was equal to 339.03 ± 42.62 mm and 181.39 ± 35.03 mm for D_F_ and D_Hy_, respectively. While, average cross-section for the false duct was equal to 580.36 ± 62.22 mm and 460.45 ± 47.94 mm for D_F_ and D_Hy_, respectively.

For P3 (Figure 1g) average value of diameter for the common duct was equal to 32.51 ± 2.96 mm and 31.92 ± 2.63 mm for D_F_ and D_Hy_, respectively. The average value of diameter for the true duct was equal to 17.93 ± 3.53 mm and 9.95 ± 2.84 mm for D_F_ (Figure 1h) and D_Hy_ (Figure 1i), respectively. Furthermore, average value of diameter for the false duct was equal to 30.70 ± 2.61 mm and 25.50 ± 2.68 mm for D_F_ and D_Hy_, respectively.

**Figure 4:**
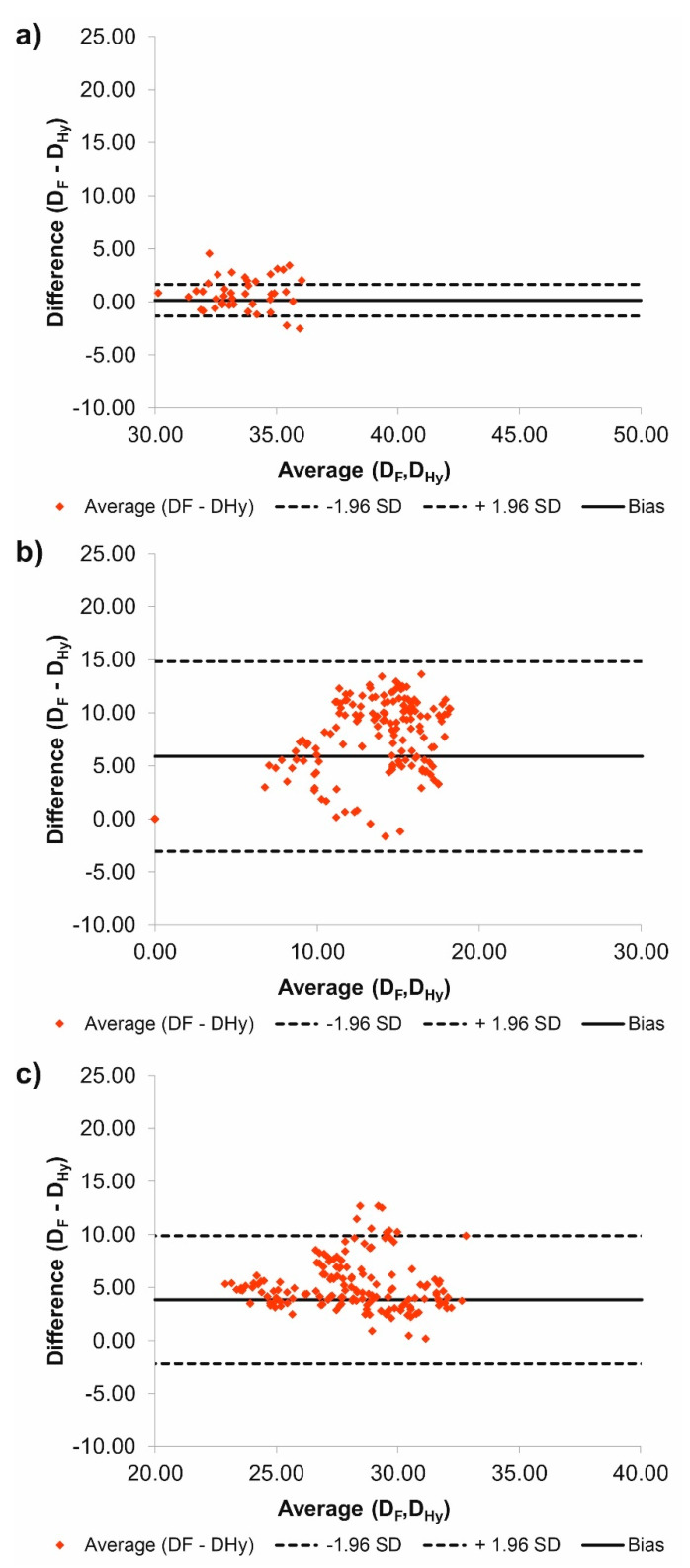
Comparison of D_F_ and D_Hy_ with the use of Bland–Altman analysis for: (**a**) Common duct, (**b**) true duct, and (**c**) false duct. For all analyzes *p* > 0.05.

Additionally, according to Bland–Altman analysis for the common duct for patient number 3 (P3) the difference between D_F_ and D_Hy_ was equal to 0.16 mm for the range equal to 2.99 mm (Figure 4a), for the true duct it was 5.88 mm for the range 17.89 mm (Figure 4b), while for the false duct it was 3.83 mm for the range 12.07 mm (Figure 4c). What is more, for the common duct 12 points stand out the optimal range, for the true duct 0 points stand out the optimal range, while for the false duct 9 points stand out the optimal range.

The average cross-section for common duct was eq The distance from descending aorta to the diaphragm was analyzed equal to 836.69 ± 147.53 mm and 816.83 ± 120.46 mm for D_F_ and D_Hy_, respectively. Moreover, average cross-section for the true duct was equal to 262.27 ± 92.29 mm and 139.82 ± 53.46 mm for D_F_ and D_Hy_, respectively. While, average cross-section for the false duct was equal to 745.58 ± 125.29 mm and 625.40 ± 106.64 mm for Feret and Hydraulic diameter, respectively.

### 3.2. Brightness Value Analysis

**Figure 5:**
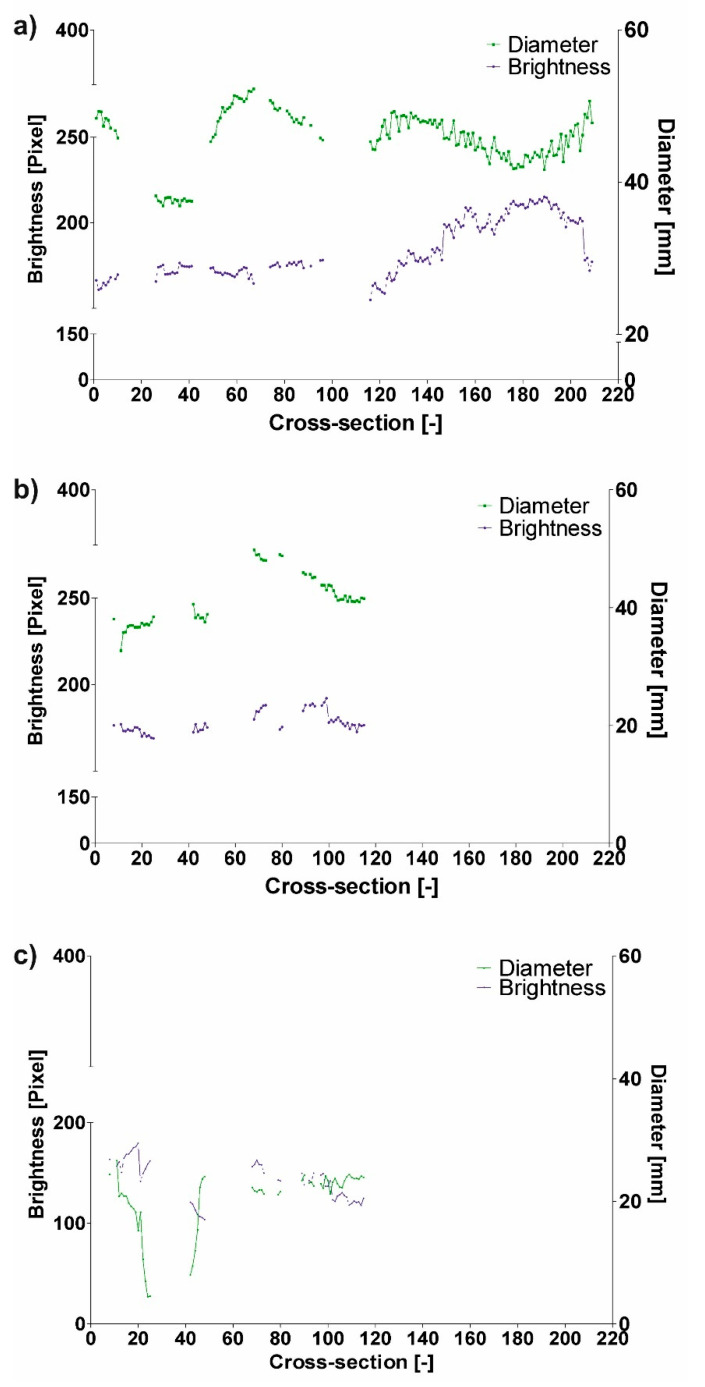
Comparison of brightness and D_F_ for patient 1 (P1): (**a**) Common duct, (**b**) true duct, and (**c**) false duct *p* > 0.05.

**Figure 6:**
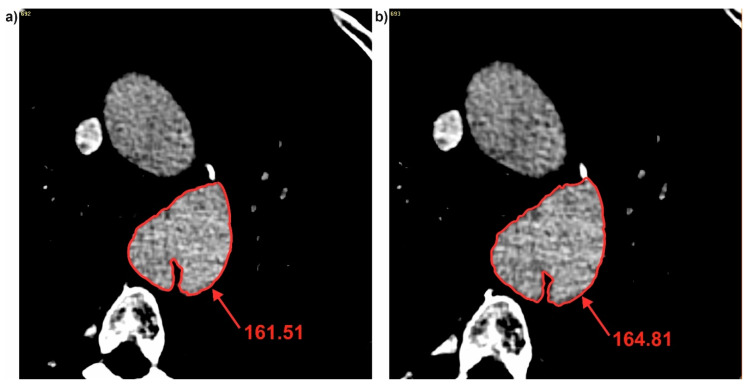
Brightness of common duct for P1: (**a**) Cross-section number 3 and (**b**) cross-section number 4. Values of brightness were calculated in Pixels.

**Figure 7:**
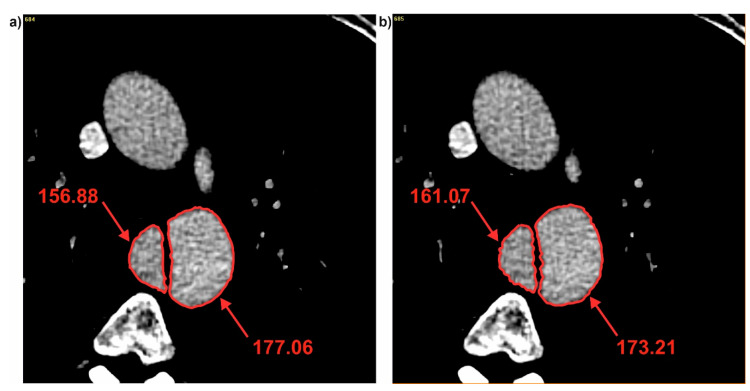
Brightness of true and false ducts for P1: (**a**) Cross-section number 11 and (**b**) cross-section number 12. Values of brightness were calculated in Pixels.

Next, brightness value for each cross-section for all three patients was analyzed. It was observed that with decrease of diameter, brightness value increased for the common duct for both D_F_ and D_Hy_ (Figure 5). For P1 decrease of diameter was from 49.27 to 47.37 (calculated for D_F_) and from 43.62 to 43.08 (calculated for D_Hy_) indicating an increase of brightness value from 161.51 to 164.81 for the common duct (for cross-section 3 and 4) (Figure 6). Similar trend was observed for the true and false ducts. Decrease of diameter for P1 for the false duct was from 26.65 to 20.80 (calculated for D_F_) and from 19.35 to 17.99 (calculated for D_Hy_) indicating an increase of brightness value from 156.88 to 161.07 (for cross-section 11 and 12) (Figure 7a). While for the false duct a decrease of diameter for P1 was from 32.70 to 35.78 (calculated for D_F_) from 33.16 to 36.50 (calculated for D_Hy_) indicating an increase of brightness value from 177.06 to 173.21 (for cross-section 11 and 12) (Figure 7b).

Additionally, average difference between the common and false duct for all three patients was equal to 13.50 ± 8.31. Meanwhile, average difference between the true and false duct was equal to 60.33 ± 34.89. Furthermore, average difference between the common and true duct was equal to 51.30 ± 23.37 (Table 3).

**Figure 8:**
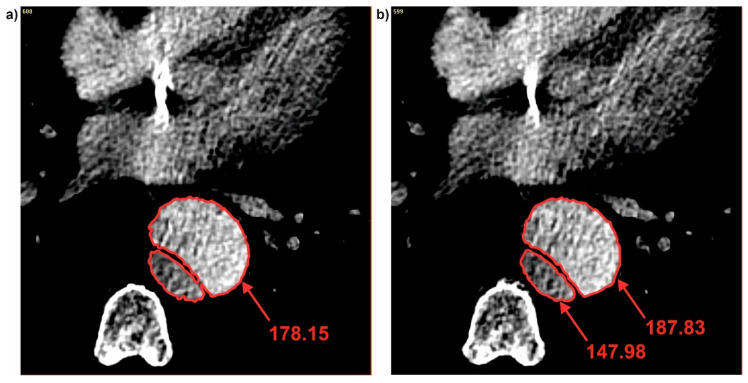
Brightness changes during channel division: (**a**) Common duct and (**b**) true and false duct. Values of brightness were calculated in pixels.

**Figure 9:**
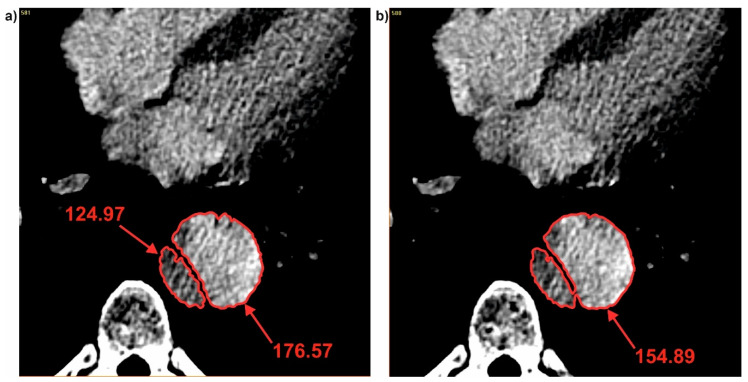
Brightness changes during channels connection: (**a**) Common duct and (**b**) true and false duct. Values of brightness were calculated in pixels.

It was also observed that each time when dissection appeared, brightness value in the true lumen was smaller compare to the common duct. While, brightness value calculated for the false lumen each time was higher compare to the common duct. For P1 when the common duct was divided into true and false (for cross-section 96 and 97) brightness value was changed from 178.15 (common duct) (Figure 8a) into 147.98 (true lumen) and 187.83 (false lumen) (Figure 8b). While, for the case when connection of the true and false duct appeared, brightness value between true and false lumen was observed. For P1 when true and false duct created common duct (for cross-section 115 and 116) brightness value was changed from 124.97 (true duct) and 176.57 (false duct) (Figure 9a) into 154.89 (common duct) (Figure 9b).

### 3.3. Difference in Brightness Value and Diameter

**Figure 10:**
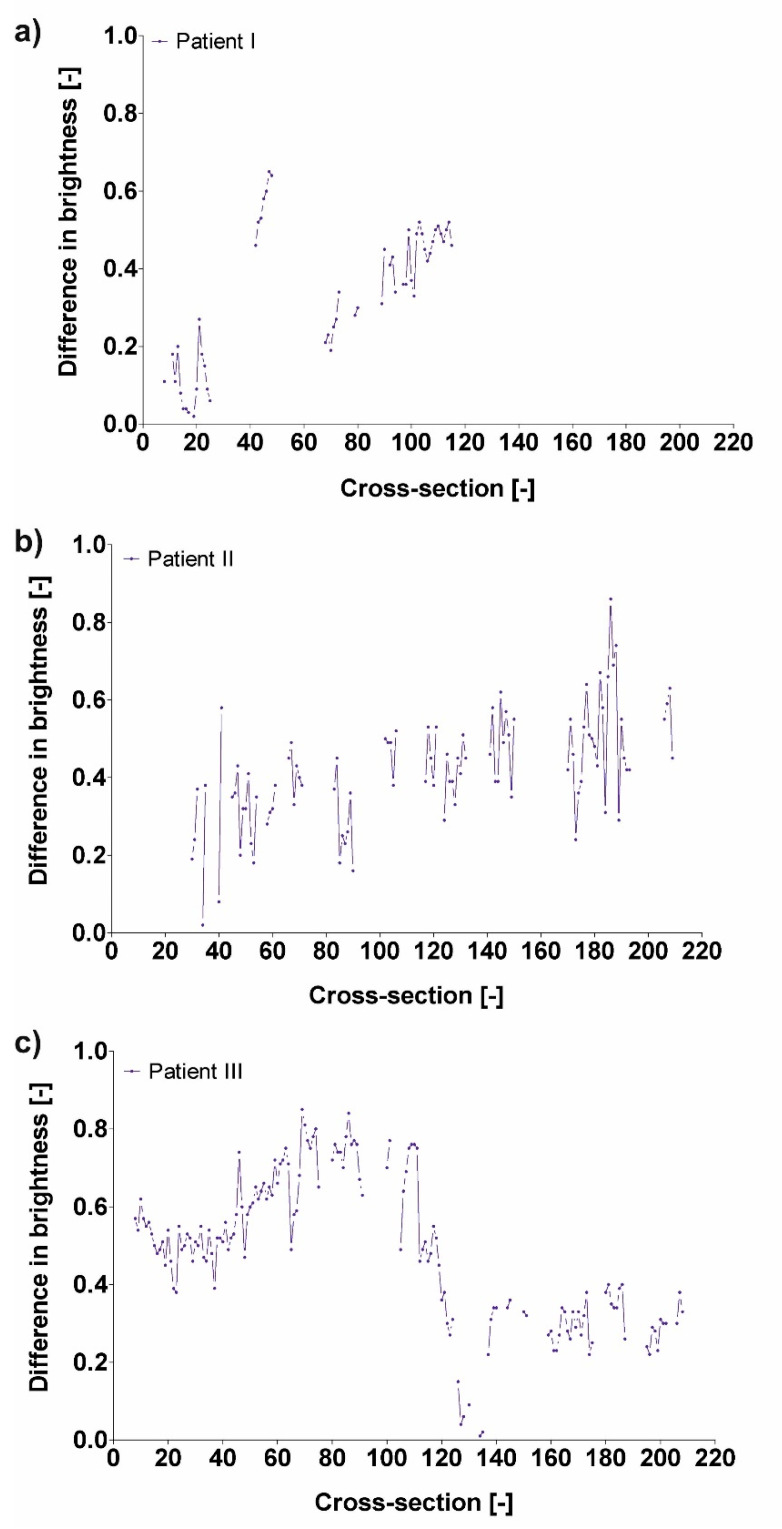
Difference in brightness value for (**a**) Patient I, (**b**) Patient II, and (**c**) Patient III.

Finally, difference in brightness value (Table 4) and diameter (Table 5 and Table 6) was analyzed. It was observed that an increase of difference in diameter indicated a decrease of difference in brightness value. For P1 increase of difference in diameter from 0.13 to 0.33 (calculated for D_F_) and from 0.40 to 0.54 (calculated for D_Hy_) indicated a decrease of difference in brightness value from 0.18 to 0.11 (for cross-section 11 and 12) (Figure 10).

## 4. Discussion

The paper presents a new computational approach to standardize the image processing technique for the virtual prediction of blood hemodynamic in the area of aortic acute type B dissection. The novelty of this paper is associated with combination of brightness and diameter analysis to detect changes in blood hemodynamics in true and false channel simultaneously. We investigated how different configuration of aortic duct affects brightness intensity which reflects blood hemodynamic in this area. In our study analysis of Feret and hydraulic diameters, enabled to estimate the effect of changes in flow conditions in the region of false and true lumen connection. Moreover, contrast enhancement analysis allowed deduction of blood hemodynamic including tearing position which may support the present model of radiological diagnosis limited to diameter analysis [30,31]. Additionally, there are situations when the pressure in both channels is equal, and then there will be no drug force and a patient may suffer from ischemia. Therefore, contrast enhancement in blood hemodynamic analysis provides information about the potential risk of such flow stagnation and for instance show places with potential risk of ischemia and/or thrombosis. It may even indicate the places of tears appearance in the intima, which is crucial and is not detected during diameter analysis. Of note, the amount of applied contrast is a constant parameter in the aorta and its concentration is a function of blood hemodynamic. Thus, both phenomena are correlated.

Analysis of spatial configuration of aortas confirmed that appearance of wall dissection had an impact on blood hemodynamic. Higher brightness was observed in the area of dissociation. It was in line with Rudenick et al. who noticed that tears existence and its size had impact on blood flow and velocity [40]. Also, Ahmed et al. indicated that a small tear decreases false lumen flow and velocity [41]. A similar approach was applied by Cheng et al. who successfully verified computational model with phase contrast magnetic resonance imaging (PC MRI) [42]. Changes in velocity profiles were based on the changes in spatial configuration of the geometry of aorta and aortic branches.

Moreover, it was noticed that higher brightness value appeared in the false ducts compare to the true ducts. It was in line with Dillon-Murphy et al. who found that during dissection around 80% of stroke volume enters the false lumen, which may further increase the dilation of the aorta [43]. Furthermore, it was recently described that changes in flow conditions influence mechanical properties of vessels [44,45,46]. Also Cheng et al. reported that high values of Wall Shear Stress around the entry tear inside the true lumen, could increase the likelihood of tear expansion [8]. Additionally, Doyle et al. observed that peak wall stress values are influenced by vessel centreline asymmetry and maximum diameter [47].

### Limitations to the Study

Although our study demonstrates the novel methodology for the description of blood hemodynamic it has some limitations. Firstly, we analyzed only acute type IIIb aortic dissection (the distance from descending aorta to the diaphragm), therefore the obtained data may not be applicable for other types of aortic dissections without initial verification. Moreover, the small sample size could influence the obtained results. However, the patients were carefully selected to uniform the group, hence we believe the obtained results may be applicable to similar cases. Secondly, simulations accuracy depends on the resolution of CTA data. The higher the resolution the better is the three-dimensional reconstruction and the final results of brightness values reconstruction. The next step would be to extend this study and analyze wider group of patients. Moreover, in present study we analyze only patients before endovascular aortic repair. We are aware that metal structures from prosthesis may affect the brightness analysis and we would like to include this parameter in our further work.

## 5. Conclusions

In summary, the performed analysis of reconstructed aortic dissection from MR images enabled visualization of dissection cross-section and brightness distribution.

Our study indicates that brightness parameter is directly connected with the dissection appearance. Each time when connection of the true duct and false duct appeared, the true duct had lower brightness compare to the common and false duct. Moreover, false duct was characterized with higher brightness compare to the common duct. Therefore, the described method may become a useful non-invasive quantitative tool for the characterization of blood hemodynamic in the area of dissection.

## Figures and Tables

**Table 1:** Spatial configuration of analyzed patients. Dissection location according to Fillinger et al. 2010 [34].

Name	Patient 1 (P1)	Patient 2 (P2)	Patient 3 (P3)
Dissection Type	IIIb	IIIb	IIIb
**Entry Tear**	Proximal to the left subclavian artery (LSA) (zone number 4 according to Fillinger et al. 2010)	Proximal to the left subclavian artery (LSA) (zone number 4 according to Fillinger et al. 2010)	Proximal to the left subclavian artery (LSA) (zone number 4 according to Fillinger et al. 2010)
**End of dissection**	Right iliac artery (zone number 9 according to Fillinger et al. 2010)	Right iliac artery (zone number 9 according to Fillinger et al. 2010)	Right iliac artery (zone number 9 according to Fillinger et al. 2010)

**Table 2:** Brightness intensity (BI) and contrast to noise ratio (CNR) for the analyzed patients.

Name	Patient 1 (P1)	Patient 2 (P2)	Patient 3 (P3)
**BI—common duct**	12.85	20.96	46.76
**BI—true duct**	8.87	28.29	8.00
**BI—false duct**	31.81	28.04	38.56
**CNR—common duct**	3.60	4.40	5.03
**CNR—true duct**	3.75	4.31	4.89
**CNR—false duct**	3.84	4.77	4.82

**Table 3:** Average brightness values measured in pixels including standard deviation (± SD).

Patient	Average Brightness		
	Common	True	False
Pat I	184.73 ± 16.75	141.36 ± 20.26	178.01 ± 6.04
Pat II	331.11 ± 18.41	364.03 ± 14.10	320.10 ± 12.60
Pat III	291.13 ± 6.60	213.52 ± 39.70	313.91 ± 8.62

**Table 4:** Average difference in brightness value included standard deviation (±SD).

Patient	Difference in Brightness
Pat I	0.33 ± 0.18
Pat II	0.42 ± 0.14
Pat III	0.48 ± 0.20

**Table 5:** Average difference in diameter for Feret diameter included standard deviation (± SD).

Patient	Difference in Diameter
Pat I	0.46 ± 0.12
Pat II	0.56 ± 0.14
Pat III	0.45 ± 0.16

**Table 6:** Average difference in diameter for hydraulic diameter included standard deviation (±SD).

Patient	Difference in Diameter
Pat I	0.58 ± 0.13
Pat II	0.57 ± 0.17
Pat III	0.59 ± 0.18

## References

[1] Hagan P.G., Nienaber C.A., Isselbacher E.M., Bruckman D., Karavite D.J., Russman P.L., Evangelista A., Fattori R., Suzuki T., Oh J.K. (2000). The International Registry of Acute Aortic Dissection (IRAD): New insights into an old disease. Jama J. Am. Med. Assoc..

[2] Roberts C.S., Roberts W.C. (1991). Aortic dissection with the entrance tear in the descending thoracic aorta. Analysis of 40 necropsy patients. Ann. Surg..

[3] Suzuki T., Mehta R.H., Ince H., Nagai R., Sakomura Y., Weber F., Sumiyoshi T., Bossone E., Trimarchi S., Cooper J.V. (2003). Clinical profiles and outcomes of acute type B aortic dissection in the current era: Lessons from the International Registry of Aortic Dissection (IRAD). Circulation.

[4] Xiong Z., Yang P., Li D., Qiu Y., Zheng T., Hu J. (2020). A computational fluid dynamics analysis of a patient with acute non-A-non-B aortic dissection after type I hybrid arch repair. Med. Eng. Phys..

[5] Ryzhakov P., Soudah E., Dialami N. (2019). Computational modeling of the fluid flow and the flexible intimal flap in type B aortic dissection via a monolithic arbitrary Lagrangian/Eulerian fluid-structure interaction model. Int. J. Numer. Methods Biomed. Eng..

[6] Polanczyk A., Podyma M., Trebinski L., Chrzastek J., Zbicinski I., Stefanczyk L. (2016). A Novel, Attempt to Standardize Results of CFD Simulations Basing on Spatial Configuration of Aortic Stent-Grafts. PLoS ONE.

[7] Polanczyk A., Podgorski M., Wozniak T., Stefanczyk L., Strzelecki M. (2018). Computational Fluid Dynamics as an Engineering Tool for the Reconstruction of Hemodynamics after Carotid Artery Stenosis Operation: A Case Study. Medicina.

[8] Cheng Z., Tan F.P., Riga C.V., Bicknell C.D., Hamady M.S., Gibbs R.G., Wood N.B., Xu X.Y. (2010). Analysis of flow patterns in a patient-specific aortic dissection model. J. Biomech. Eng..

[9] Polanczyk A., Podgorski M., Polanczyk M., Piechota-Polanczyk A., Stefanczyk L., Strzelecki M. (2019). A novel vision-based system for quantitative analysis of abdominal aortic aneurysm deformation. Biomed. Eng. Online.

[10] Kociolek M., Strzelecki M., Klepazko A. (2019). Functional Kidney Analysis Based on Textured DCE-MRI Images. Adv. Intell. Syst. Comput..

[11] Polanczyk A., Podgorski M., Polanczyk M., Piechota-Polanczyk A., Neumayer C., Stefanczyk L. (2018). A Novel Patient-Specific Human Cardiovascular System Phantom (HCSP) for Reconstructions of Pulsatile Blood Hemodynamic Inside Abdominal Aortic Aneurysm. IEEE Access.

[12] Tyfa Z., Witkowski D., Sobczak K., Obidowski D., Jozwik K. (2018). Experimental investigations of the aerated polymethylmethacrylate-based vertebral cement flow in capillaries. Int. J. Artif. Organs.

[13] Polanczyk A., Strzelecki M., Wozniak T., Szubert W., Stefanczyk L. (2017). 3D Blood Vessels Reconstruction Based on Segmented CT Data for Further Simulations of Hemodynamic in Human Artery Branches. Found. Comput. Decis. Sci..

[14] Polanczyk A., Podyma M., Stefanczyk L., Zbicinski I. (2012). Effects of stent-graft geometry and blood hematocrit on hemodynamic in Abdominal Aortic Aneurysm. Chem. Process Eng..

[15] Bessonov N., Sequeira A., Simakov S., Vassilevskii Y., Volpert V. (2016). Methods of blood flow modeling. Math. Model. Nat. Phenom..

[16] Hoi Y., Meng H., Woodward S.H., Bendok B.R., Hanel R.A., Guterman L.R., Hopkins L.N. (2004). Effects of arterial geometry on aneurysm growth: Three-dimensional computational fluid dynamics study. J. Neurosurg..

[17] Cloutier G., Zimmer A., Yu F.T., Chiasson J.L. (2008). Increased shear rate resistance and fastest kinetics of erythrocyte aggregation in diabetes measured with ultrasound. Diabetes Care.

[18] Chi Q., He Y., Luan Y., Qin K., Mu L. (2017). Numerical analysis of wall shear stress in ascending aorta before tearing in type A aortic dissection. Comput. Biol. Med..

[19] Polanczyk A., Podyma M., Stefanczyk L., Szubert W., Zbicinski I. (2015). A 3D model of thrombus formation in a stent-graft after implantation in the abdominal aorta. J. Biomech..

[20] Polanczyk A., Wozniak T., Strzelecki M., Szubert W., Stefanczyk L. Evaluating an algorithm for 3D reconstruction of blood vessels for further simulations of hemodynamic in human artery branches. Proceedings of the 2016 Signal Processing: Algorithms, Architectures, Arrangements, and Applications (SPA).

[21] Janke D., Jankowski J., Ruth M., Buschmann I., Lemke H.D., Jacobi D., Knaus P., Spindler E., Zidek W., Lehmann K. (2013). The “artificial artery” as in vitro perfusion model. PLoS ONE.

[22] Thomas A., Daniel Ou-Yang H., Lowe-Krentz L., Muzykantov V.R., Liu Y. (2016). Biomimetic channel modeling local vascular dynamics of pro-inflammatory endothelial changes. Biomicrofluidics.

[23] Campbell B.C., Christensen S., Levi C.R., Desmond P.M., Donnan G.A., Davis S.M., Parsons M.W. (2012). Comparison of computed tomography perfusion and magnetic resonance imaging perfusion-diffusion mismatch in ischemic stroke. Stroke.

[24] Chen Y., Zhang Y., Yang J., Cao Q., Yang G., Chen J., Shu H., Luo L., Coatrieux J.L., Feng Q. (2016). Curve-Like Structure Extraction Using Minimal Path Propagation With Backtracking. IEEE Trans. Image Process..

[25] Hirano T. (2014). Searching for salvageable brain: The detection of ischemic penumbra using various imaging modalities?. J. Stroke Cerebrovasc. Dis. Off. J. Natl. Stroke Assoc..

[26] Obuchowicz R., Piorkowski A., Urbanik A., Strzelecki M. (2019). Influence of Acquisition, Time on MR Image Quality Estimated with Nonparametric Measures Based on Texture Features. Biomed Res. Int..

[27] Tyfa Z., Obidowski D., Jozwik K. (2018). Numerical analysis of the VAD outflow cannula positioning on the blood flow in the patient–specific brain supplying arteries. Mech. Mech. Eng..

[28] Iannaccone F., De Beule M., Verhegghe B., Segers P. (2013). Computer Simulations in Stroke Prevention: Design Tools and Virtual Strategies Towards Procedure Planning. Cardiovasc. Eng. Technol..

[29] Wardlaw J.M., Sandercock P.A., Berge E. (2003). Thrombolytic therapy with recombinant tissue plasminogen activator for acute ischemic stroke: Where do we go from here? A cumulative meta-analysis. Stroke.

[30] Kim H.Y., Yong H.S., Kim E.J., Kang E.Y., Seo B.K. (2018). Value of transluminal attenuation gradient of stress CCTA for diagnosis of haemodynamically significant coronary artery stenosis using wide-area detector CT in patients with coronary artery disease: Comparison with stress perfusion CMR. Cardiovasc. J. Afr..

[31] Fujimoto S., Giannopoulos A.A., Kumamaru K.K., Matsumori R., Tang A., Kato E., Kawaguchi Y., Takamura K., Miyauchi K., Daida H. (2018). The transluminal attenuation gradient in coronary CT angiography for the detection of hemodynamically significant disease: Can all arteries be treated equally?. Br. J. Radiol..

[32] Polanczyk A., Piechota-Polanczyk A., Christoph D., Nanobachvili J., Huk I., Neumayer C. (2018). Computational Fluid Dynamic Accuracy in Mimicking Changes in Blood Hemodynamics in Patients with Acute Type IIIb Aortic Dissection Treated with TEVAR. Appl. Sci. Basel.

[33] Polanczyk A., Piechota-Polanczyk A., Neumayer C., Huk I., Gutschmidt S., Hewett J., Sellier M. (2019). CFD reconstruction of blood hemodynamic based on a self-made algorithm in patients with acute type IIIb aortic dissection treated with TEVAR procedure. IUTAM Symposium on Recent Advances in Moving Boundary Problems in Mechanics.

[34] Fillinger M.F., Greenberg R.K., McKinsey J.F., Chaikof E.L. (2010). Society for Vascular Surgery Ad Hoc Committee on TRS. Reporting standards for thoracic endovascular aortic repair (TEVAR). J. Vasc. Surg..

[35] Polanczyk A., Podgorski M., Polanczyk M., Veshkina N., Zbicinski I., Stefanczyk L., Neumayer C. (2019). A novel method for describing biomechanical properties of the aortic wall based on the three-dimensional fluid-structure interaction model. Interact. Cardiovasc. Thorac. Surg..

[36] Assi A.A.N., Arra A.A. (2017). Optimization of image quality in pulmonary CT angiography with low dose of contrast material. Pol. J. Med Phys. Eng..

[37] Polanczyk A., Piechota-Polanczyk A., Stefanczyk L. (2017). A new approach for the pre-clinical optimization of a spatial configuration of bifurcated endovascular prosthesis placed in abdominal aortic aneurysms. PLoS ONE.

[38] Harris C., Alcock A., Trefan L., Nuttall D., Evans S.T., Maguire S., Kemp A.M. (2018). Optimising the measurement of bruises in children across conventional and cross polarized images using segmentation analysis techniques in Image, J.; Photoshop and circle diameter measurements. J. Forensic Leg. Med..

[39] Sahar M.A., Wissink J., Mahmoud M., Karayiannis T.G., Ishak M.A. (2017). Effect of hydraulic diameter and aspect ratio on single phase flow and heat transfer in a rectangular microchannel. Appl. Therm. Eng..

[40] Rudenick P.A., Bijnens B.H., Garcia-Dorado D., Evangelista A. (2013). An in vitro phantom study on the influence of tear size and configuration on the hemodynamics of the lumina in chronic type B aortic dissections. J. Vasc. Surg..

[41] Ben Ahmed S., Dillon-Murphy D., Figueroa C.A. (2016). Computational Study of Anatomical Risk Factors in Idealized Models of Type B Aortic Dissection. Eur. J. Vasc. Endovasc. Surg. Off. J. Eur. Soc. Vasc. Surg..

[42] Cheng Z., Juli C., Wood N.B., Gibbs R.G., Xu X.Y. (2014). Predicting flow in aortic dissection: Comparison of computational model with PC-MRI velocity measurements. Med Eng. Phys..

[43] Dillon-Murphy D., Noorani A., Nordsletten D., Figueroa C.A. (2016). Multi-modality image-based computational analysis of haemodynamics in aortic dissection. Biomech. Modeling Mechanobiol..

[44] Pintoux D., Chaillou P., Azema L., Bizouarn P., Costargent A., Patra P., Gouëffic Y. (2011). Long-term influence of suprarenal or infrarenal fixation on proximal neck dilatation and stentgraft migration after EVAR. Ann. Vasc. Surg..

[45] Valencia A., Campos F., Munizaga J., Perez J., Rivera R., Bravo E. (2011). Hemodynamics in cerebral aneurysms models and the effects of a simple stent models. Adv. Appl. Fluid Mech..

[46] Poon E.K., Barlis P., Moore S., Pan W.H., Liu Y., Ye Y., Xue Y., Zhu S.J., Ooi A.S. (2014). Numerical investigations of the haemodynamic changes associated with stent malapposition in an idealised coronary artery. J. Biomech..

[47] Doyle B.J., Callanan A., Burke P.E., Grace P.A., Walsh M.T., Vorp D.A., McGloughlin T.M. (2009). Vessel asymmetry as an additional diagnostic tool in the assessment of abdominal aortic aneurysms. J. Vasc. Surg..

